# New Orleans Clinics

**Published:** 1892-12

**Authors:** Robert Morris

**Affiliations:** Resident Student in Charity Hospital


For Daniel's Texas Medical Journal.

### NEW ORLEANS CLINICS.

[Reported by Robert Morris, Resident Student in Charity Hospital.]

---

### AORTIC ANEURISM AND PHTHISIS PULMONALIS.

Charles Williams, black, aet. 36, entered Ward 31, Charity Hospital, August 6, '92.

He contracten syphilis in 1881 or '82. The manifestations being various; general eruption, alopecia, and mucous patches. In 1889 he experienced shooting pains in the chest, and noticed that he swallowed with difficulty. At the same time he became hoarse, which hoarseness increased until he was unable to talk above a whisper. In '91 he noticed a swelling on right side of sternum, which gradually increased in size.

A physical examination revealed the following:

*Inspection*—A tumor about the size of a hen's egg in second intercostal space to right of sternum, which visibly pulsated.

*Palpation* — Increased vocal fremitus over right lung. The hand when applied over the tension rose and fell synchronously with the heart's action. The right radial pulse was absent.

*Percussion*—Dullness over tissue around the tumor, also diminished dullness over both lungs.

*Auscultation*—A double blow at the apex of the heart was heard but none at the base. No bruit was heard.

*Rales*—Subcrepitant rales were heard at the apex of left lung and scattered over the whole of right lung.

*Voice Sounds*—The vocal resonance was slightly increased at apex of left lung, bronchophonic over right lung, except over a small area in infra-clavicular region where whispering pectoriloquy was heard.

*Respiratory Sounds*—The respiration was cavernous in character over the area where the pectoriloquy was heard. The expiratory sound was prolonged over both lungs.

*Treatment*—The iodide of potassium was given in 10 gr. doses, three times per day, which afforded some relief. Its efficacy is due, I think, to the dilitation of the peripheral blood vessels, thus diminishing the pressure in the large arteries, and in this particular case, the pressure in the aneurism. This diminished intra-vascular pressure, and slowered circulation, favored the deposition of fibrine in the aneurismal sac. The iodide of potassium also increased bronchial secretion, thus relieving the patient of the irritative cough due to the pressure of the aneurism. The usual remedies were given for his pulmonary trouble, such as cod-liver oil and hypophosphites. His fever was septic in character and treated by cold baths. The patient sank rapidly, and died August 30th, 1892.

The post mortem showed a tumor, about 5 in. in diameter, in middle mediastinal space, which was firmly adherent to second rib on right, and surrounding tissue. The trachea and bronchi were closely connected to the tumor. There was pressure on the œsophagus and right recurrent laryngeal nerve. The aneurism involved the ascending and transverse part of the arch of the aorta primarily and the innominate secondarily. The heart was large and flabby.

Fully $2\frac{1}{2}$ in. of fibrin in the thickness was seen when sac was opened, which was in beautiful layers, each layer possessing its pecular color due to its age and consistency. The cavity was very small, about one inch in circumference, which accounted satisfactorily for the abscence of the bruit. Each lung was tuberculous and adherent; the right containing a cavity about the size of a hen's egg.

This case illustrates the close relationship existing between syphilis and aneurism; how endortaritis, produced by syphilis, will so weaken the vessel walls, that they, under the slightest provocation, will dilate, and eventually an aneurism is produced. It furthermore shows how environment, and those causes which interfere with the proper nourishment of the lung, as pressure from an aneurism, favor the deposition and growth of the bacilli tuberculosis. He claimed that there was no hereditary tendency to phthisis, and that up to the time he contracted syphilis, he was strong and hearty.

FEMORAL ANEURISM.

The patient, Sport Darling, black, age 38, entered ward 3, Charity Hospital, Oct. 31, 1892. He was shot three years ago with a pistol, the ball entering midway the thigh on its outer aspect, making its exit to the inner side of a line drawn from the center of Poupart's ligament to inner condyle of femur.

The wound healed readily, leaving a swelling about the size of a pigeon's egg, which moved to and fro, as he expressed it. This tumor increased slowly in size until Oct. 27th, 1892, when he fell over the tongue of a wagon, bruising the inner part of wounded thigh. Immediately, the tumor enlarged and continued to increase in size until his entrance in the hospital, Oct. 31, 1892, when it was the size of a croquet ball, and visibly pulsated.

Inasmuch as the tumor had increased very rapidly since Oct. 27th, an operation was decided upon, and patient conveyed to operating room. The haste in operating was due to the fact that the man's life was in danger, either from hemorrhage or gangrene. Patient was anæsthetized with chloroform. The usual antiseptic precautions were observed, and an Esmarc was applied from the toe to above the aneurism. Prof. Logan, the surgeon in charge, made a liberal incision over most prominent part of tumor and removed considerable febrin and freshly coagulated blood. The incision was enlarged and explored, and eight or nine pieces of bone were removed, varying in size from a dime to a quarter. The bone, no doubt, was injured by the ball, and a spicula penetrated the vessel wall, which ultimately produced the aneurism. Both ends of artery were securely ligated, the sac was cleansed with a solution of bichloride of mercury, 1-3000, and packed with gauze. It was then closed with three retentive sutures. He was sent to ward, where hot applications were placed around the limb. He made an uneventful recovery; the collateral circulation being sufficient to maintain the integrity of the limb.

This case is especially interesting when we remember that the bone was injured three years ago, and that no untoward symptoms arose which could be attributed to dead bone, although several particles of bone were completely detached.

